# Tracing cellular heterogeneity in pooled genetic screens via multi-level barcoding

**DOI:** 10.1186/s12864-019-5480-0

**Published:** 2019-02-06

**Authors:** Michael Boettcher, Sergio Covarrubias, Anne Biton, James Blau, Haopeng Wang, Noah Zaitlen, Michael T. McManus

**Affiliations:** 1 Department of Microbiology and Immunology, UCSF Diabetes Center, University of California, San Francisco, San Francisco, CA 94143 USA; 2 Department of Medicine, Lung Biology Center, University of California, San Francisco, San Francisco, 94143 CA USA; 3 Institut Pasteur, Hub Bioinformatique et Biostatistique, Centre de Bioinformatique, Biostatistique et Biologie Intégrative (C3BI, USR 3756 Institut Pasteur et CNRS), Paris, France; 4 Departments of Medicine and of Microbiology & Immunology, the Rosalind Russell-Ephraim P. Engleman Medical Research Center for Arthritis, and the Howard Hughes Medical Institute, University of California, San Francisco, San Francisco, CA 94143 USA

**Keywords:** CRISPR, Screening, Genome editing, Clonal heterogeneity

## Abstract

**Background:**

While pooled loss- and gain-of-function CRISPR screening approaches have become increasingly popular to systematically investigate mammalian gene function, the large majority of them have thus far not investigated the influence of cellular heterogeneity on screen results. Instead most screens are analyzed by averaging the abundance of perturbed cells from a bulk population of cells.

**Results:**

Here we developed multi-level barcoded sgRNA libraries to trace multiple clonal Cas9 cell lines exposed to the same environment. The first level of barcoding allows monitoring growth kinetics and treatment responses of multiplexed clonal cell lines under identical conditions while the second level enables in-sample replication and tracing of sub-clonal lineages of cells expressing the same sgRNA.

**Conclusion:**

Using our approach, we illustrate how heterogeneity in growth kinetics and treatment response of clonal cell lines impairs the results of pooled genetic screens.

**Electronic supplementary material:**

The online version of this article (10.1186/s12864-019-5480-0) contains supplementary material, which is available to authorized users.

## Background

Pooled genetic screens are a powerful tool to functionally dissect genetic networks in mammalian cells and in conjunction with recently developed CRISPR/Cas systems, they permit a variety of scalable genetic perturbations, including gene knockout, knockdown or activation [1, 2]. While numerous pooled CRISPR screens have been conducted successfully in the past, they frequently disregard a fundamental property of cell populations – namely their genotypic and phenotypic heterogeneity [3]. As a matter of fact, most CRISPR screens published to date were conducted in clonal Cas9 lines derived from single cells [4–9]. When present, replicates were mostly technical replicates as opposed to biological replicates, and processed in different pools, thereby making it impossible to investigate the effects of identical screening conditions on different clonal lines.

To enable the study of screening replicates exposed to the same conditions and dissect the influence of cellular heterogeneity on the results of pooled genetic screens, we developed a multi-level barcoding strategy for pooled single guide RNA (sgRNA) libraries. Our approach is based on the combination of every sgRNA sequence in the library with a constant library identifier (ID, level 1) and a random barcode nucleotide sequence (BC, level 2). The ID consists of a sequence that is unique to each sgRNA library within which each sgRNA is associated with a set of randomly generated BC sequences. Consequently, the ID allows the tracing of defined clonal Cas9 lines in a pool over the course of the screen. The BC on the other hand, allows the analysis of sub-clonal lineages of cells expressing a certain sgRNA sequence, similar to recently described random sequence labels [10] or unique molecular identifiers [11].

We used multi-level barcoded sgRNA libraries for genome-scale CRISPR-mediated gene knockout (CRISPRwt) as well as knockdown (CRISPRi) screens to systematically identify genes involved in TNF-related apoptosis-inducing ligand (TRAIL) mediated apoptosis [12]. Using our barcoding approach to study clonal lines with different sensitivities to TRAIL receptor (TRAIL-R) antibody, we were able to capture heterogeneity in the growth rate and treatment response of clonal Cas9 cell lines and to show differences in statistical power and thereby detectable candidate genes. Similar issues caused by clonal heterogeneity likely bias the outcomes of many genetic screens. Here we illustrate cellular heterogeneity in pooled genetic screens performed under identical conditions by means of multi-level barcoding.

## Methods

### Clonal Jurkat cell lines

Clonal Jurkat CRISPRwt and CRISPRi lines were kindly provided by Haopeng Wang [13], they were derived as previously described [13] and cultured in RPMI 1640 medium, supplemented with 10% fetal bovine serum and 1x Anti-Anti (Gibco).

### Sytox apoptosis assay

Jukat cells were treated with TRAIL receptor antibody (MAB631, R&D Systems) for indicated periods of time. Cells were then stained with a 1:500 dilution of Sytox Green (Thermofisher, S7020) and analyzed on a flow cytometer to quantify the fraction of GFP+ (dead) cells (Additional file 1: Figure S1).

### CRISPRwt and CRISPRi sgRNA library design

For both CRISPR technologies, a separate genome-scale sgRNA library was designed, each consisting of over 250,000 total sgRNAs targeting every unique Refseq annotated (hg19) protein coding isoform with up to 12 sgRNAs, plus 7700 non-target control sequences (NTC). For the CRISPRwt sgRNA library, the earliest possible exon of each transcript variant was targeted. For the CRISPRi sgRNA library, sgRNAs were targeted 50 to 500 bp downstream of the transcription start site (TSS) of each isoform. All sgRNAs were designed against target sites that are of the format (N)_20_NGG, and selected sgRNAs must pass the following off-targeting criteria: 1) the 11 bp-seed must not have an exact match in any other promoter region, and 2) if there is an exact off-target seed match, then the rest of the sgRNA must have at least 7 mismatches with the potential off-target site. After all sgRNAs that pass off-targeting criteria were generated, up to 12 sgRNAs/transcript were selected. All sgRNA sequences are shown in Additional file 2: Table S6 (CRISPRwt) and Additional file 3: Table S7 (CRISPRi).

In addition to the sgRNA sequence, every library plasmid contained two extra features that allowed us to address heritable clonal heterogeneity in CRISPR screens: 1.) A specific 6 nucleotide long library identifier (ID) sequence (IDs for CRISPRwt libraries were ‘GCCTAA’ or ‘TGGTCA’, and for CRISPRi libraries ‘CGTGAT’ or ‘ACATCG’ respectively) to allow tracing of clonal lines in a pool cells. One resistant (CloneR) and one sensitive (CloneS) clonal lines were used within each CRISPR system. And 2.) a unique 20 nucleotide barcode sequence to facilitate the analysis of sub-clonal populations (see Additional file 4 for vector map).

### sgRNA library cloning

For both the CRISPRwt and CRISPRi libraries, the designed 20 nt target specific sgRNA sequences were synthesised as a pool, on microarray surfaces (CustomArray, Inc.), flanked by overhangs compatible with Gibson Assembly into the pSico based barcoded sgLenti sgRNA library vector (see Additional file 4 for vector map). The synthesised sgRNA template sequences were of the format: 5’-GGAGAACCACCTTGTTGG-(N)_20_-GTTTAAGAGCTATGCTGGAAAC-3′. Template pools were PCR amplified using Phusion Flash High-Fidelity PCR Master Mix (ThermoFisher Scientific) according to the manufacturers protocol with 1 ng/μL sgRNA template DNA, 1 μM forward primer (5’-GGAGAACCACCTTGTTGG-3′), 1 μM reverse primer (5′- GTTTCCAGCATAGCTCTTAAAC-3′) and the following cycle numbers: 1x (98C for 3 min), 15x (98C for 1 s, 55C for 15 s, 72C for 20 s) and 1x (72C for 5 min). PCR products were purified using Minelute columns (Qiagen). The library vector sgLenti was prepared by restriction digest with AarI (Thermo-Fischer) at 37C overnight, followed by 1% agarose gel excision of the digested band and purification via NucleoSpin columns (Macherey-Nagel). Using Gibson Assmbly Master Mix (NEB), 1000 ng digested sgLenti and 100 ng amplified sgRNA library insert were assembled in a total 200 μL reaction volume. The reaction was purified using P-30 buffer exchange columns (Biorad) that were equilibrated 5x with H_2_O and the total eluted volume was transformed into three vials of Electromax DH5α (ThermoFisher). E.coli were recovered, cultured overnight in 500 mL LB (100 μg/mL ampicillin) and used for Maxiprep (Qiagen). In parallel, a fraction of the transformation reaction was plated and used to determine the total number of transformed clones. The library cloning coverage (number of E.coli colonies per sgRNA plasmid) was determined to be >100x for each of the four libraries, ensuring even representation of all library sgRNA sequences and their narrow distribution as well as the required barcode diversity for each sgRNA sequence to facilitate the tracing of sub-clonal populations.

### Lentivirus production

HEK293T cells were seeded at 65,000 cells per ccm in 15 cm dishes in 20 mL media (DMEM, 10% fetal bovine serum) and incubated overnight at 37C, 5% CO_2_. The next morning, 8 μg sgRNA library plasmid, 4 μg psPAX2 (Addgene #12260), 4 μg pMD2.G (Addgene #12259) and 40 μL jetPRIME (Polyplus) were mixed into 1 mL serum free OptiMEM (Gibco) with 1x jetPRIME buffer, vortexed and incubated for 10 min at RT and added to the cells. 24 h later, 40 U DNAseI (NEB) were added to each plate in order to remove untransfected plasmid and at 72 h post-transfection, supernatant was harvested, passed through 0.45 μm filters (Millipore, Stericup) and aliquots were stored at -80C.

### Genome-wide CRISPRwt/CRISPRi screens

Two clonal CRISPRwt and two CRISPRi Jurkat lines were transduced separately with their respective barcoded sgRNA libraries at low multiplicity of infection (MOI = 0.3) to reduce the frequency of multiple-infected cells; thus, only one gene was targeted for knockout or knockdown in each cell. The library coverage at transduction was determined to be > 100 transduced cells for each sgRNA from each of the four libraries, to ensure full representation of library sgRNA sequences in the target cell populations as well as a sufficient diversity of barcode sequences per sgRNA to facilitate sub-clonal population analyses. The four transduced clonal lines were then cultured separately in RPMI with 10% FBS and 1x Anti-Anti (Gibco) in a 37 °C incubator with 5%CO_2_. 2 days post transduction, cells were selected for 5 days with puromycin (2 μg/mL). Following puromycin selection, 1 billion cells from each of the four clonal CRISPRwt/i lines were combined. The cell pool was seeded into 2 × 5 l RPMI medium in CelliGen BLU bioreactor vessels (25 RPM, 37 °C, pH = 7.4, O_2_ = 8%) at a final density of 300,000 cells per ml - or a total of 1.5 billion cells per bioreactor - to achieve a representation of > 1000 cells per sgRNA in each of the four libraries. The remaining pool of 1 billion cells was cryo-preserved in 90% FBS, 10% DMSO for later analyses (Baseline). After pooling, cells were either treated with escalating concentrations of TRAIL receptor antibody (MAB631, R&D Systems) on day 0 (10 ng/ml), day 1 (20 ng/ml) and day 4 (25 ng/ml) or left untreated (control cells). The culture was diluted with fresh medium when exceeding a density of 1 mio cells/ml medium and a library coverage of >1000x was maintained throughout the screen to ensure equal representation of sgRNAs and barcodes. Identical to the baseline sample, aliquots of 1 billion cells from treated and untreated bioreactors were cryo-preserved on days 4, 9 and 14 for later analysis.

### Genomic DNA (gDNA) extraction

Cell pellets from baseline and imatinib treated samples were resuspended in 20 mL P1 buffer (Qiagen) with 100 μg/mL RNase A and 0.5% SDS followed by incubation at 37C for 30 min. After that, Proteinase K was added (100 μg/mL final) followed by incubation at 55C for 30 min. After digest, samples were homogenised by passing them three times through a 18G needle followed by three times through a 22G needle. Homogenised samples were mixed with 20 mL Phenol:Chlorophorm:Isoamyl Alcohol (Invitrogen #15593–031), transferred into 50 mL MaXtract tubes (Qiagen) and thoroughly mixed. Samples were then centrifuged at 1500 g for 5 min at room temperature (RT). The aqueous phase was transferred into ultracentrifuge tubes and thoroughly mixed with 2 mL 3 M sodium acetate plus 16 mL isopropanol at RT before centrifugation at 15,000 g for 15 min. The gDNA pellets were carefully washed with 10 mL 70% ethanol and dried at 37C. Dry pellets were resuspended in H_2_O and gDNA concentration was adjusted to 1 μg/uL. The degree of gDNA shearing was assessed on a 1% agarose gel and gDNA was sheared further by boiling at 95C until average size was between 10 and 20 kb.

### PCR recovery of sgRNA sequences from gDNA

Multiple PCR reactions were prepared to allow amplification of the total harvested gDNA from a 1000x cell coverage for each sample. For the first round of two nested PCRs, the total volume was 100 μL containing 50 μg sheared gDNA, 0.3 μM forward (5′-ggcttggatttctataacttcgtatagca-3) and reverse (5′-cggggactgtgggcgatgtg-3′) primer, 200 μM each dNTP, 1x Titanium Taq buffer and 1 μL Titanium Taq (Clontech). PCR cycles were: 1x (94C - 3 min), 16x (94C - 30 s, 65C – 10 s, 72C – 20 s), 1x (68C – 2 min). All first round PCRs were pooled and a fraction was used as template for the second round PCR. The total volume of the second round PCR was 100 μL containing 2 μL pooled first round PCR, 0.5 μM forward (5’-AATGATACGGCGACCACCGAGATCCACAAAAGGAAACTCACCCTAAC-3′) and reverse (5’-CAAGCAGAAGACGGCATACGAGAT-(N)6-GTGACTGGAGTTCAGACGTG-3′) primer where (N)_6_ is a 6 nt index for sequencing on the Illumina HiSeq platform, 200 μM each dNTP, 1x Titanium Taq buffer and 1 μL Titanium Taq (Clontech). PCR cycles were: 1x (94C - 3 min), 16x (94C - 30 s, 55C – 10 s, 72C – 20 s), 1x (68C – 2 min). The resulting PCR product (344 bp) was extracted from a 1% agarose gel. Gel extracted bands were submitted for sequencing on an Illumina HiSeq 2500 platform using paired end 50 kits with the custom sequencing primer 5’-GAGACTATAAGTATCCCTTGGAGAACCACCTTGTTGG-3′ for reading the sgRNA sequence and the standard Truseq Illumina reverse primer to read out 20 nt unique barcode sequences and library IDs.

### Sequencing reads preprocessing

The sgRNA, library IDs and BC sequence information obtained through paired-end next generation sequencing was extracted from every read sequence using a python script (https://github.com/quasiben/gRNA_Tool). Reads with the same combination of sgRNA sequence, library IDs, and BC sequence were summed up together to generate a BC-based count matrix for each library-ID.

### Barcode built-in replicates and analysis of the CRIPSR screens

Barcodes of a same sgRNA that were within a Hamming distance of one were collapsed. If several overlapping pairs of barcodes were within a Hamming distance of one, we only kept the barcode with the largest count and ignored the others. Barcodes with less than 5 read counts in at least one sample were discarded. 12,800,918 sgRNA-barcode pairs were selected for CRISPRwt CloneS, 10,403,933 for CRISPRwt CloneR, 11,4106,76 for CRISPRi CloneS, and 3,975,262 for CRISPRi CloneR.

Barcodes were then randomly split into three groups for each sgRNA. Read counts of the barcodes belonging to a same group were summed up and used as one built-in replicate. Only sgRNAs for which at least 3 barcodes were detected were included in the analysis. In order to keep sgRNAs with a number of read counts sufficient to be used for built-in replication and the MAGeCK analysis, we discarded the sgRNAs with less than 5 read counts in any of the built-in replicates at day 14. This led to the selection of 120,711 sgRNAs for CloneS of CRISPRwt, 211,742 sgRNAs for CloneR of CRISPRwt, 155,895 sgRNAs for CloneS of CRISPRi, and 158,642 sgRNAs for CloneR of CRISPRi. These sgRNAs were targeting 28,361 and 36,253 RefSeq genes for CRISPRwt in CloneS and CloneR, respectively; and 18,634 and 18,666 RefSeq genes for CRISPRi in CloneS and CloneR respectively.

The three built-in replicates were used as input for MAGeCK v0.5.5, with default parameters to detect sgRNAs that were positively enriched in the TRAIL-treated samples for each clonal population, each CRISPR system, and each time point independently.

### Barcode subsampling to explore cell coverage

For each selected sgRNA with at least four barcodes detected at baseline, we randomly sub-sampled different proportions of the barcodes (from 5 to 100% of the barcodes, increasing by step of 5%) and performed the MAGeCK analysis on each of the sets of sub-sampled data. Barcodes were summed up for each sgRNA and days 9 and 14 were used as replicates since built-in replicates could not be used because of small number of available barcodes that could be obtained when small proportions of subsampling were used. Only sgRNAs with at least four barcodes were used and, when needed, proportions were rounded to the closest proportion that could be obtained with the minimum number of barcodes. Each subsampling was iterated 50 times, and the obtained gene ranks were averaged across the 50 iterations for each subsampling proportion.

## Results

### Generation of clonal Cas9 lines

**Fig. 1:**
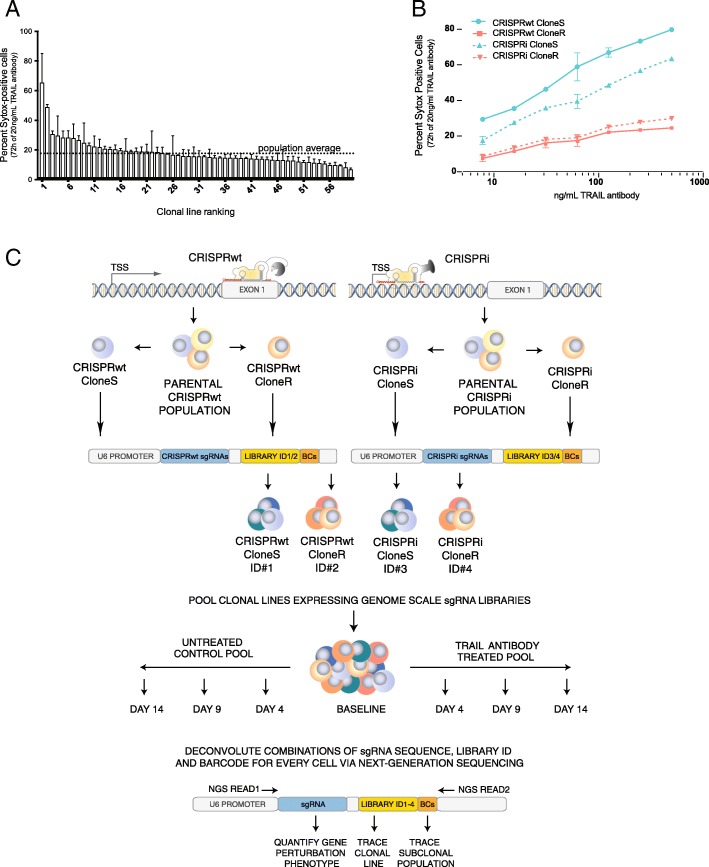
**a** Apoptosis response of clonal Cas9 Jurkat cell lines following 72 h treatment with 20 ng/μL TRAIL-R antibody, as assessed by Sytox staining and FACS. Standard deviation obtained from duplicate experiments is shown. Gating strategy for assessing Sytox is shown in Additional file 1: Figure S1. **b** Apoptosis response of the four clonal Cas9 lines used for pooled CRISPR screens at indicated concentrations of TRAIL-R antibody. Standard deviation is shown for duplicate experiments. **c** Schematic of clonal heterogeneity CRISPR screening and deconvolution approach. For CRISPRwt (top left) and CRISPRi (top right) the respective Cas9 gene was introduced into a population of Jurkat cells followed by the characterization of clonal lines for functionality of each CRISPR system. From both systems, two clonal lines were transduced with a multi-level barcoded sgRNA library (12 sgRNAs/gene) to knockout (CRISPRwt) or knockdown (CRISPRi) each protein coding gene in the human genome. Successfully transduced cells were pooled at equal numbers and the abundance of each clonal lines was traced via one of four library identifier sequences (ID) throughout the screen. Cell pools were cultured for 14 days in the absence (bottom left) or presence (bottom right) of TRAIL-R antibody. For downstream analysis via next-generation sequencing, sgRNA expression cassettes including the sgRNA encoding sequence, ID and BCs were recovered via PCR from the genomic DNA of cell pools from the beginning of the screen (baseline) as wells as on days 4, 9 and 14. Using a paired-end sequencing strategy allowed the quantification of the dis−/enrichment of sub-clonal populations (BC) within clonal lines (library ID) following the perturbation of any protein-coding gene in the human genome (sgRNA sequence)

Either Cas9 nuclease (CRISPRwt) or dCas9-KRAB (CRISPRi) was introduced into a population of Jurkat T lymphocytes. Clonal lines from either system were expanded and the function of their respective CRISPR systems was confirmed as previously described [13]. Although all clonal lines were derived from the same parental population, they displayed substantial variability in their response to TRAIL-R antibody treatment (Fig. 1a). To investigate the impact of the observed heterogeneity on CRISPR screens, one resistant (CloneR) and one sensitive clone (CloneS) from either CRISPR system was used for pooled screens to identify genes involved in TRAIL-mediated apoptosis (Fig. 1b).

### Multi-level barcoded CRISPR screen

Each clonal line was transduced with one of four barcoded sgRNA libraries, targeting every protein coding gene in the human genome for knockout (CRISPRwt) or knockdown (CRISPRi). To enable tracing of the relative abundance of the clonal Cas9 lines, each sgRNA library was tagged with one of four different IDs. After lentiviral transduction of the sgRNA libraries at low MOI, followed by the selection of successfully transduced cells, the four clonal lines were mixed at equal numbers, ensuring a representation of each sgRNA in over 1000 cells from each Cas9 line. The cells were then split into two bioreactor-vessels: one untreated control vessel and one vessel treated with escalating doses of TRAIL-R antibody on days 0, 2 and 4. Cells from the beginning of the screen (baseline) as well as from days 4, 9 and 14 were harvested from both vessels. ID, BC and sgRNA sequences from each time point were recovered via PCR and quantified by means of paired-end next generation sequencing (Fig. 1c).

### Library IDs can trace clonal heterogeneity

**Fig. 2:**
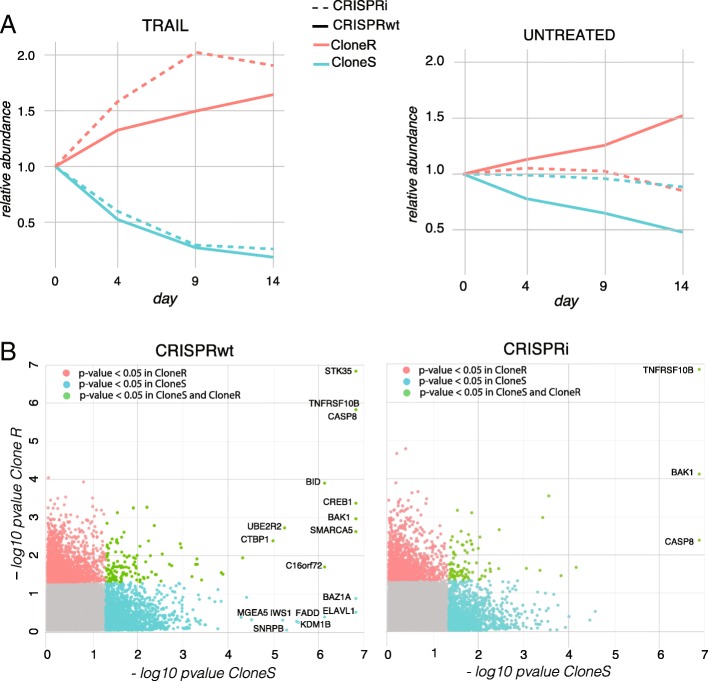
**a** Relative abundance of clonal CRISPRwt and CRISPRi populations in the TRAIL-receptor antibody treated cell pool (left) and in the untreated pool (right). Abundance of IDs normalized to the baseline (day 0) is shown on the y-axis. **b** Screen results summary. The –log_10_ *p*-values obtained from MAGeCK analysis are shown. Genes highlighted in green have p-values below 0.05 in both clones, genes in blue have p-values lower than 0.05 only in CloneS only, and genes in red in CloneR only. Names of genes with a FDR < 5% are indicated

The unique sgRNA library IDs allowed us to follow the relative abundance of the four clonal lines over time in both pools. For the two resistant clones, enrichment was observed following TRAIL-R antibody treatment, as shown by an increase in the fraction of sequencing reads mapping to IDs of the resistant clonal populations while both sensitive clones became depleted over time (Fig. 2a left panel and Additional file 5: Table S1). To a lesser extent, differences in proliferation rates of the untreated cell clones were observed between both clonal wtCas9 cell lines (Fig. 2a, right panel). Those results confirm the aforementioned heterogeneous apoptosis response of the four clonal Cas9 lines (Fig. 1b) and suggest that the clonal lines used for the screen do not only display heterogeneity in response to TRAIL-R antibody treatment but also in proliferation rates. Taken together, those results illustrate the suitability of library ID sequences to trace clonal lines in pooled cell populations.

### CRISPR screen analysis

In order to identify genes involved in TRAIL-mediated apoptosis, all four clonal screens were analyzed using MAGeCK [14]. Untreated and treated samples were compared within each clonal line and each CRISPR system. Due to the heterogeneous TRAIL-R antibody responses observed between the clonal Cas9 lines, an increasing number of reads mapped to sgRNAs expressed in the resistant versus the sensitive clonal lines (Additional file 5: Table S1). The lowest number of total read counts observed was 20 million reads for the sensitive CRISPRwt clone after 14 days of TRAIL-R antibody treatment. While this read depth is on the lower end for negative selection screens, it allowed us to detect genes whose perturbation caused cells to enrich after 14 days of TRAIL-R antibody treatment. All other clonal lines showed substantially higher read depths (Additional file 5: Table S1).

A higher number of significantly enriched candidate genes (FDR < 5%) was detected in the CRISPRwt compared to CRISPRi screens (Fig. 2b, Additional file 6: Table S2-S5). These results are consistent with the claim that CRISPRwt outperforms CRISPRi in identifying essential genes [8]. In addition, we found a higher number of significantly enriched candidate genes in sensitive versus the resistant clones. This finding is most likely explained by the fact that the TRAIL-R antibody concentration used in the screen, was optimal for the selection of the sensitive but sub-optimal for resistant clones, leading to low levels of correlation between clonal lines despite identical experimental conditions (Additional file 7: Figure S2, panel A). Stronger selection of the sensitive clones resulted in stronger relative enrichment of cells expressing sgRNAs targeting candidate genes when compared to resistant clones, as it is for instance the case for the TRAIL receptor TNFRSF10B (Additional file 7: Figure S2, panel B). TNFRSF10B, BAK1, and CASP8 were found as significantly enriched in all clonal populations. The distributions of the barcode fold-changes for the sgRNAs targeting these three candidate genes illustrates the level of heterogeneity in the response of sub-clonal populations carrying the same sgRNA (Additional file 8: Figure S3).

### Use of barcodes for in-sample replication and study of sub-clonal variation

**Fig. 3:**
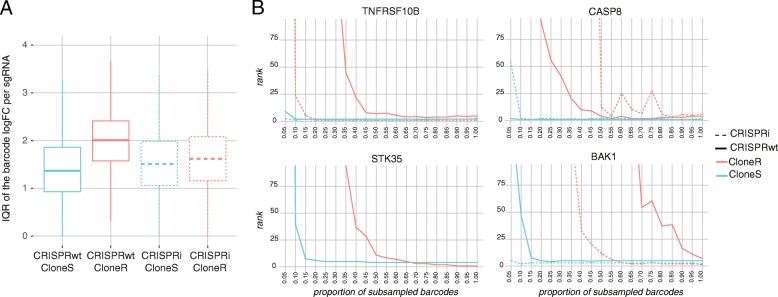
**a** The distribution of the dispersion of the barcode log fold changes (TRAIL-R antibody treated vs untreated) at day 14 for each sgRNA is shown for each of the four clonal Cas9 lines. For each sgRNA, the interquartile range (IQR) of the barcodes log fold-changes was computed. **b** Barcode subsampling across the four clonal Cas9 lines. The x-axis shows the fraction of barcodes sampled for each sgRNA. The y-axis shows the rank of four genes (A) TNFRSF10B, B) CASP8, C) STK35, D) BAK1) in the MAGeCK output obtained from the subsampled datasets

Next, we explored the utility of random barcodes (BCs) associated with each sgRNA sequence (Additional file 9: Figure S4, panels A and B) to (i) derive screen replicates, (ii) trace individual sub-clonal populations and (iii) simulate the impact of reduced screen complexity on screen results. We first studied whether sgRNA-barcode pairs were reproducible across time points of the untreated condition to make sure that the dataset was likely not impacted by PCR template switching previously described [15, 16] (Additional file 10: Figure S5). More than 98% of the sgRNA-barcode pairs with large read counts at day 14 were also detected at day 4. As expected, sgRNA-barcode pairs with lower read counts were recovered at a lower rate (> 75%). The proportions of sgRNA-barcode pairs detected at both day 14 and baseline were slightly lower, likely because of the differences in representation of both clonal and cellular populations before and after mixing the populations in a new culture vessel. Despite the changes in clonal population across time, these percentages remained high and confirmed that template switching, if present, was minimal. Then, all BCs were randomly split into three bins, providing three in-sample replicates (Additional file 9: Figure S4, panel C) that were then used as input for MAGeCK analysis. High levels of correlation between in-sample technical replicates confirm that the randomly assembled sub-populations within each clonal Cas9 line showed an overall similar behavior (Additional file 9: Figure S4, panel D). Sub-clonal heterogeneity can be quantified by studying differences in response to TRAIL across cells carrying a same sgRNA using the barcodes. Figure 3a shows the distribution of the dispersion of the BC log fold changes across sgRNAs. The resistant clone showed a larger dispersion of the effect of TRAIL-R compared to the sensitive clone within each CRISPR system (Wilcoxon test *p*-values < 10^− 12^). These results suggest that the effect of the differences in selection pressure undergone by the two clones is also visible at the sub-clonal level where higher levels of sub-clonal heterogeneity in response to TRAIL-R antibody treatment is observed in the resistant compared to the sensitive clones.

Two major concerns for every pooled CRISPR screen are (i) sgRNA library representation in the target cells and (ii) sgRNA sequence read depth. For screens conducted with large sgRNA libraries or in difficult to culture cells, these two issues can rapidly become limiting factors and in some scenarios, such as in-vivo screens, it is even impossible to determine sgRNA library representation. Hence assessing and reducing the complexity of genetic screens, without compromising statistical power is critical to success in many challenging screen setups. To investigate the effects of reduced complexity on the screen results, we sub-sampled fractions of BCs associated with each sgRNA in the library. Since each sgRNA-BC sequence combination represents a unique lentiviral integration event, and with that a distinctive sub-clonal population, subsampling the BCs allowed us to retrospectively investigate the effects of reduced screen complexity on screen results. We determined the minimum fraction of BCs required to significantly call genes identified from the full read counts. As shown in Fig. 3b on the example of the candidate genes TNFRSF10B (TRAIL receptor), CASP8, BAK1, and STK35 (only significantly enriched in CRISPRwt), only 25% or less of the sequencing reads were sufficient to identify all candidate genes in CloneS of the CRISPRwt screen while in CloneR over 60% of the reads were necessary to call even the strongest hits TNFRSF10B, CASP8 and STK35. Similar results were obtained from both clones of the CRISPRi screens. These findings illustrate how clonal Cas9 lines with heterogeneous levels of resistance require different levels of screen complexity to capture a similar enrichment signal. More importantly, these results confirm the utility of BCs to determine the level of saturation in genetic screens.

## Discussion

CRISPR screens are typically conducted in clonal cell lines derived from a single Cas9 expressing cell and thus do not capture the full clonal heterogeneity found in the parental populations they derive from. Screening the bulk of parental cells on the other hand can mask results obtained from more sensitive sub-clonal populations. Here we present a multi-level barcoding approach to follow individual clonal as well as sub-clonal cell populations in pooled CRISPR screens under identical conditions. Using this combinatorial barcoding approach, we were able to individually trace four pooled clonal populations over the course of a CRISPR screen. We demonstrate how clonal heterogeneity can affect statistical power to detect candidate genes when clonal lines are cultured and selected under identical conditions. Our study shows that the same dose of TRAIL-R antibody treatment can lead to very different CRISPR screen outcomes from different clonal Cas9 cell lines obtained from the same parental population, despite high levels of technical reproducibility. These findings argue for future CRISPR screens to be conducted in multiple clonal lines in parallel rather than just technical replicates of the same clonal Cas9 line.

## Conclusions

We developed a strategy for multi-level barcoding of sgRNA libraries and exemplify its utility by performing a genetic screen using two different CRISPR systems to detect known and novel regulators of TRAIL-mediated apoptosis in co-cultures of several clonal T-cell lines. This multi-level barcoding approach can be incorporated into any type of pooled library to permit parallel genetic screens in co-cultures, as well as the analysis of their distinctive sub-populations.

## Additional files

Additional file 1: **Figure S1.** FACS plots from the first column show gated cells (gate1) that were included in the sytox analysis. Column 2 FACS plots show sytox positive cells (gate 2). The last column shows the concentration of TRAIL used for the FACS plot in each row. As expected, higher TRAIL Antibody results in higher sytox staining. (PDF 658 kb)

Additional file 2: **Table S6.** CRISPRwt sgRNA library sequences. (XLSX 17092 kb)

Additional file 3: **Table S7.** CRISPRi sgRNA library sequences. (XLSX 11476 kb)

Additional file 4 Vector map.ᅟ(GB 18 kb)

Additional file 5: **Table S1.** Number of read counts mapped to each clonal population within each CRISPR system. (XLSX 54 kb)

Additional file 6: **Tables S2-S5.** MAGeCK outputs at the gene-level from CRISPRwt CloneR (Table S2), CRISPRi CloneS (Table S3), CRISPRi CloneR (Table S4) and CRISPRwt CloneS (Table S5). Columns are described at https://sourceforge.net/p/mageck/wiki/output/#gene_summary_txt. (ZIP 35431 kb)

Additional file 7: **Figure S2.** A. Upper panel. Scatter plots of the gene log2 fold changes of the TRAIL condition against the untreated condition between the sensitive (CloneS, x-axis) and the resistant clone (CloneR, y-axis) at day 14, for CRISPRwt (left) and CRISPRi (right). Lower panel. Scatter plots of the gene log2 fold changes of the TRAIL condition against the untreated condition between CRISPRwt (x-axis) and CRISPRi (y-axis) for the sensitive (CloneS, left) and the resistant clones (CloneR, right) at day 14. B. Mean-difference (MD) plots at the sgRNA level. Y-axis shows the sgRNA log2 fold change of the TRAIL-treated condition over the untreated condition. X-axis shows the sgRNA average log2 read count. Random sgRNAs are shown in light grey, sgRNAs of five positive controls (CASP8, TNFRSF10B, CREB1, FADD, STK35, BAK1) are superimposed in different colors, with a triangle shape if the sgRNA gets a FDR lower than 5% in the MAGeCK analysis. (PDF 29669 kb)

Additional file 8: **Figure S3.** Distribution of the log2 fold changes (TRAIL over untreated, day 14) across barcodes of the sgRNAs targeting known markers of TRAIL-R mediated apoptosis. Each boxplot shows the distribution of the log2 fold changes (TRAIL over untreated) of all barcodes of a given sgRNA at day 14 in one clone. Each dot represents one of the barcode log2 ratio. The resistant clone (CloneR) data are shown in red, while the sensitive clone data (CloneS) are shown in blue. Only barcodes with at least 5 counts in both untreated and treated samples at day 14 are represented. (PDF 1369 kb)

Additional file 9: **Figure S4.** A. Distribution of the (log2) number of barcodes per sgRNA. B. Plot of the density of barcode log2 read count distribution per clonal population and CRISPR system. C. Plot of the density of log2 read count distribution of the three built-in replicates for each clonal population and CRISPR system. D. Scatter plots of sgRNA normalized log2 read counts, at baseline (day 0), between three built-in replicates made by randomly splitting barcodes into three groups for each sgRNA. Spearman rank correlation values between replicates are indicated. (PDF 6104 kb)

Additional file 10: **Figure S5.** Reproducibility of the sgRNA-barcode pairs across time points of the untreated condition. The x-axis shows 10 bins of sgRNA-barcode pairs that were built based on the quantiles of their log2 read counts distribution at day 14 for each clonal population in the untreated condition. The y-axis shows the proportion of pairs within each bin that were also detected (at least one read count) at baseline/day0, day 4, and day 9. (PDF 7 kb)

## Abbreviations

CloneR: Resistant clone
CloneS: Sensitive clone
CRISPRi: dCas9-KRAB based CRISPR interference
CRISPRwt: Cas9 nuclease based CRISPR
FDR: False discovery rate
IQR: Interquartile Range
sgRNA: single guide RNA
TRAIL: TNF-related apoptosis-inducing ligand
TRAIL-R: TRAIL receptor
