# Supplementary figures and images for: Tracing cellular heterogeneity in pooled genetic screens via multi-level barcoding
Source: BMC Genomics. 2019 Feb 6;20:107. doi: 10.1186/s12864-019-5480-0 (PMC6364396; doi:10.1186/s12864-019-5480-0)

Column:

I

II

III

**TRAIL Ab**  
0 ng/ml

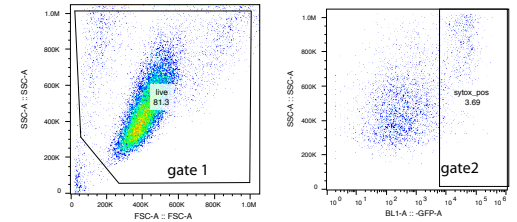

8 ng/ml

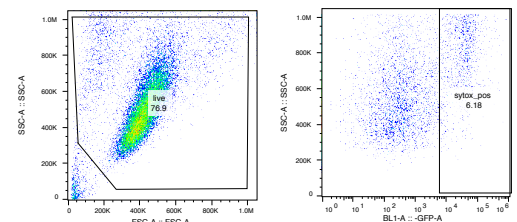

16 ng/ml

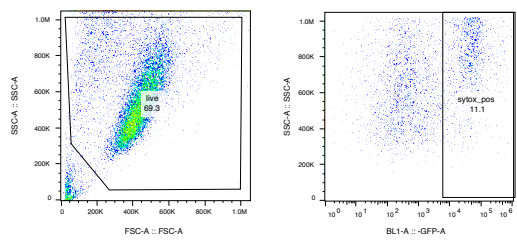

31 ng/ml

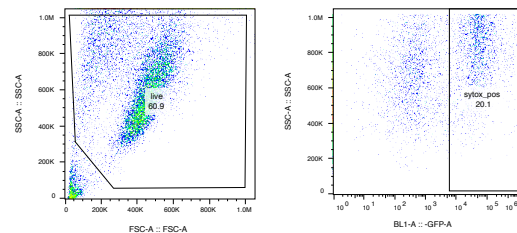

62 ng/ml

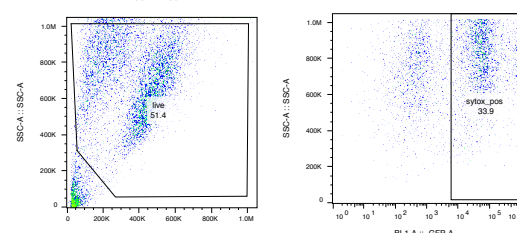

125 ng/ml

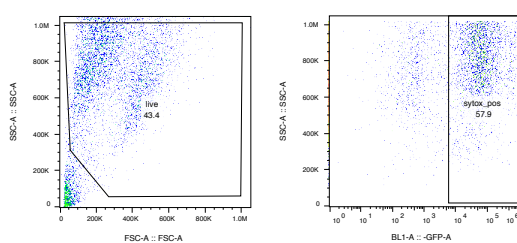

250 ng/ml

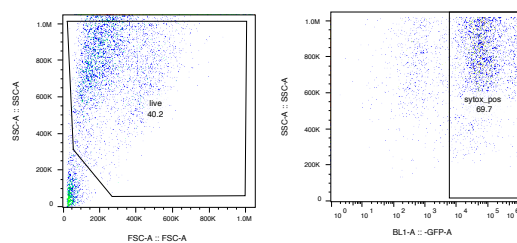

500 ng/ml

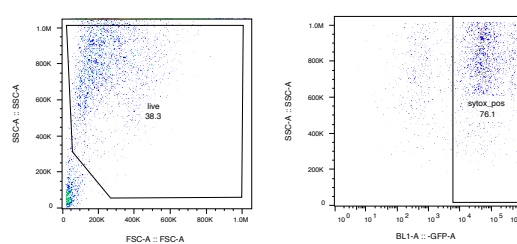

Supplement: Supplementary file 1 — Figure S1. FACS plots from the first column show gated cells (gate1) that were included in the sytox analysis. Column 2 FACS plots show sytox positive cells (gate 2). The last column shows the concentration of TRAIL used for the FACS plot in each row. As expected, higher TRAIL Antibody results in higher sytox staining. (PDF 658 kb) [file 12864_2019_5480_MOESM1_ESM.pdf]

A

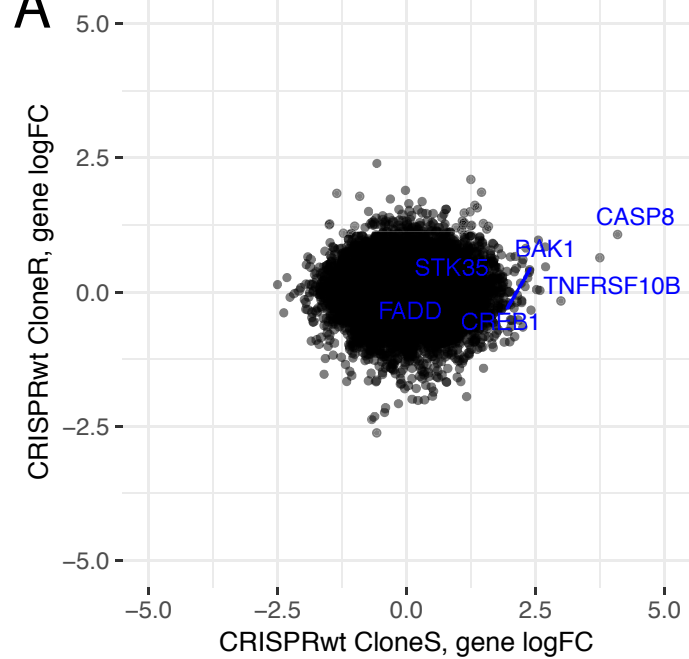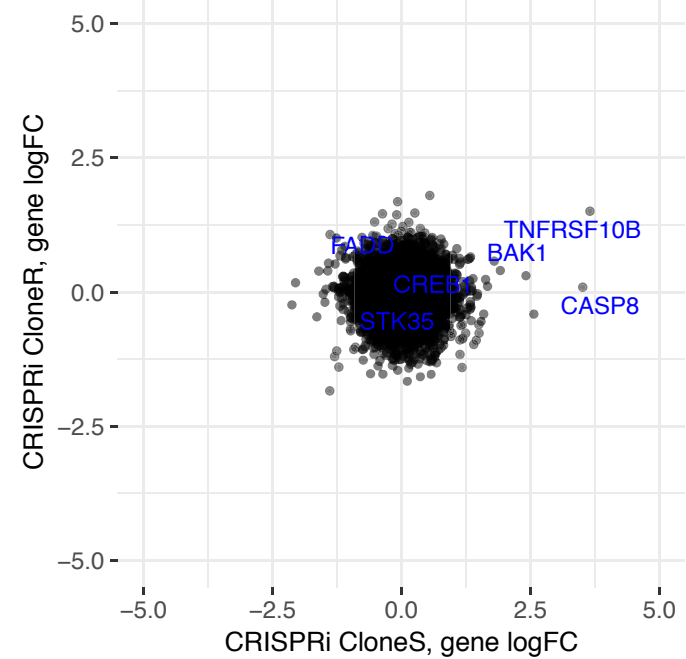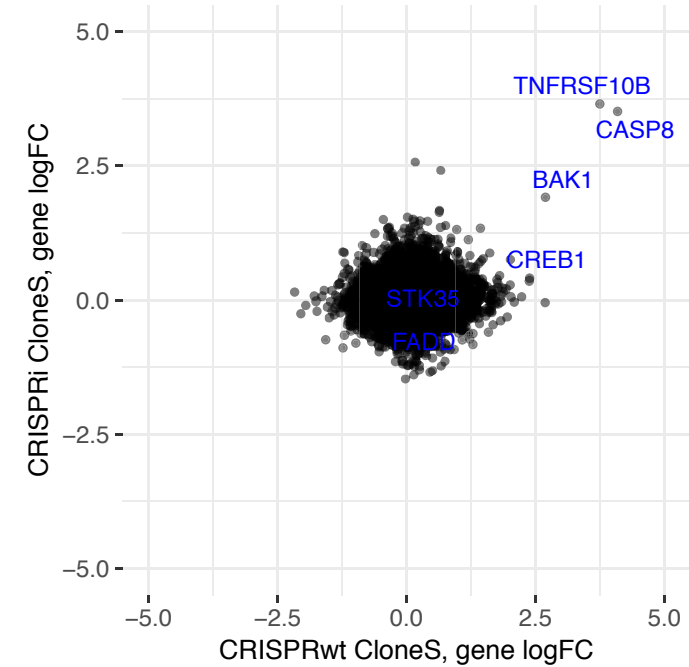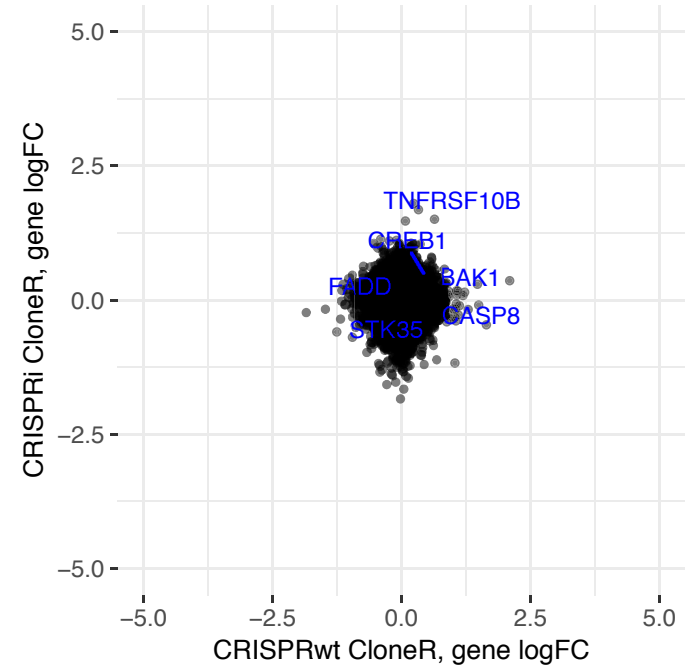

# B

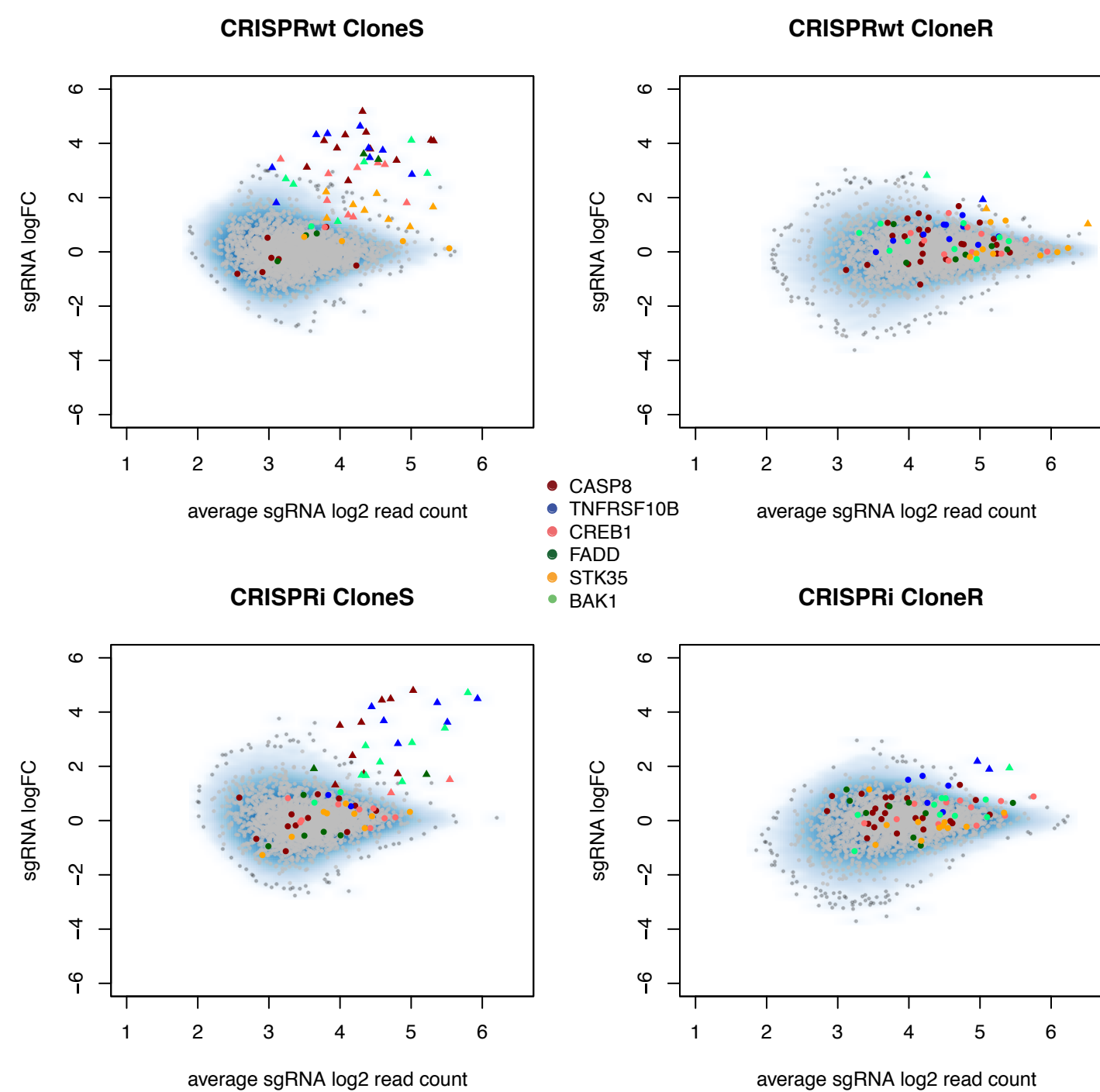

Supplement: Supplementary file 7 — Figure S2. A. Upper panel. Scatter plots of the gene log2 fold changes of the TRAIL condition against the untreated condition between the sensitive (CloneS, x-axis) and the resistant clone (CloneR, y-axis) at day 14, for CRISPRwt (left) and CRISPRi (right). Lower panel. Scatter plots of the gene log2 fold changes of the TRAIL condition against the untreated condition between CRISPRwt (x-axis) and CRISPRi (y-axis) for the sensitive (CloneS, left) and the resistant clones (CloneR, right) at day 14. B. Mean-difference (MD) plots at the sgRNA level. Y-axis shows the sgRNA log2 fold change of the TRAIL-treated condition over the untreated condition. X-axis shows the sgRNA average log2 read count. Random sgRNAs are shown in light grey, sgRNAs of five positive controls (CASP8, TNFRSF10B, CREB1, FADD, STK35, BAK1) are superimposed in different colors, with a triangle shape if the sgRNA gets a FDR lower than 5% in the MAGeCK analysis. (PDF 29669 kb) [file 12864_2019_5480_MOESM7_ESM.pdf]

CRISPRwt

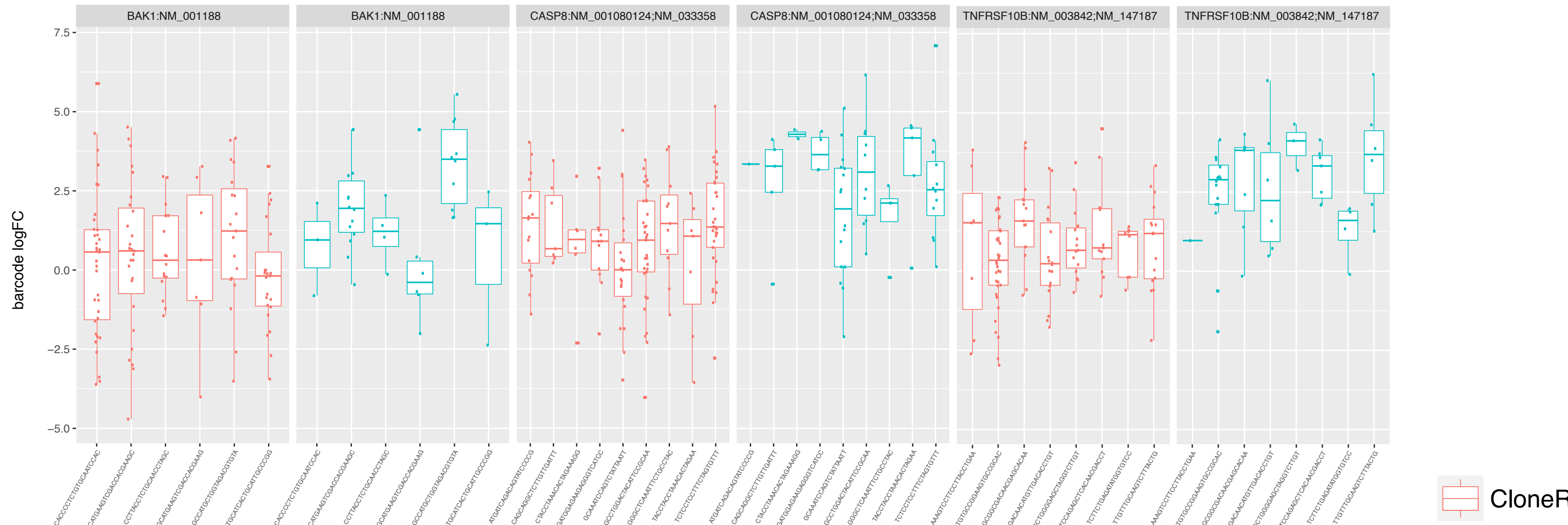

CRISPRi

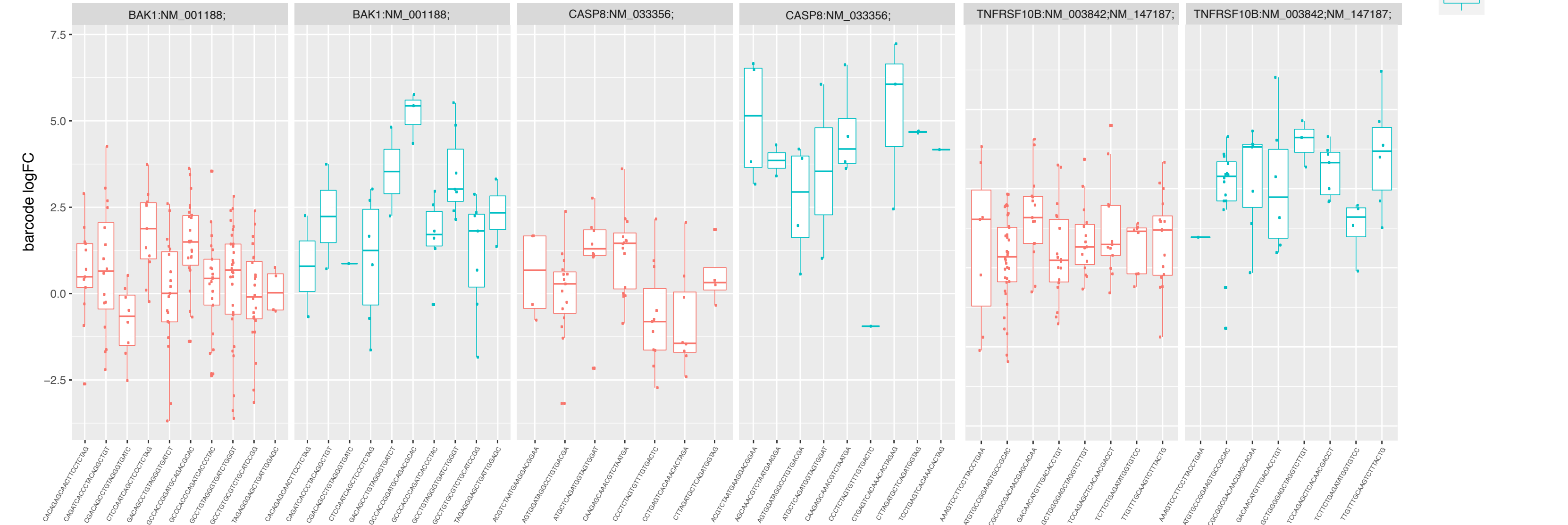

sgRNA

Supplement: Supplementary file 8 — Figure S3. Distribution of the log2 fold changes (TRAIL over untreated, day 14) across barcodes of the sgRNAs targeting known markers of TRAIL-R mediated apoptosis. Each boxplot shows the distribution of the log2 fold changes (TRAIL over untreated) of all barcodes of a given sgRNA at day 14 in one clone. Each dot represents one of the barcode log2 ratio. The resistant clone (CloneR) data are shown in red, while the sensitive clone data (CloneS) are shown in blue. Only barcodes with at least 5 counts in both untreated and treated samples at day 14 are represented. (PDF 1369 kb) [file 12864_2019_5480_MOESM8_ESM.pdf]

CRISPRi Cloner

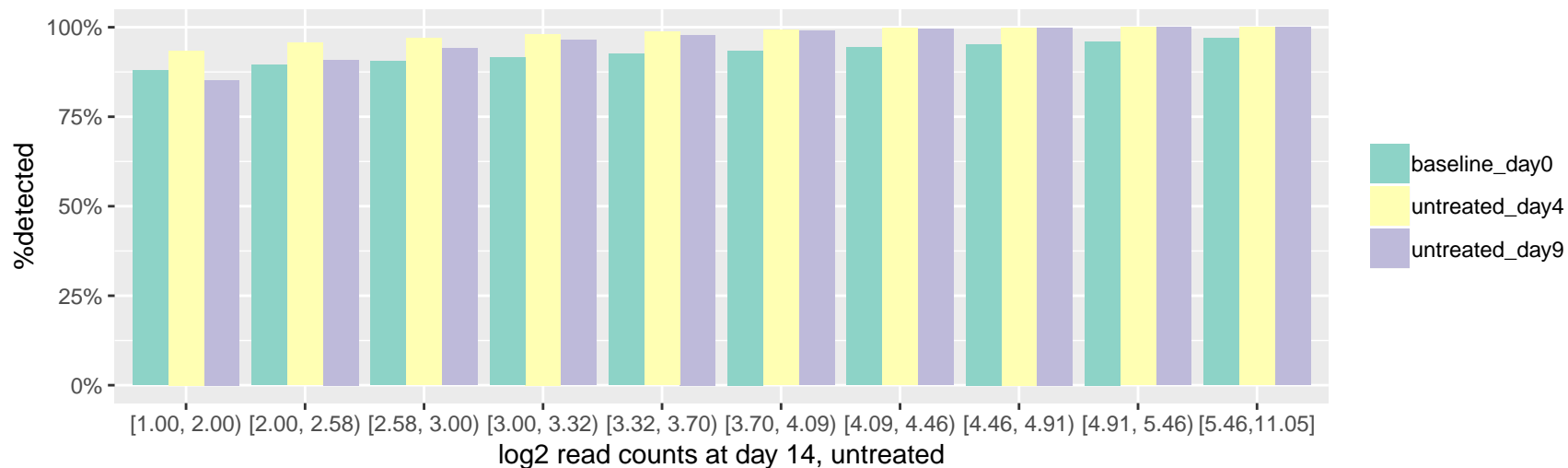

CRISPRi Clones

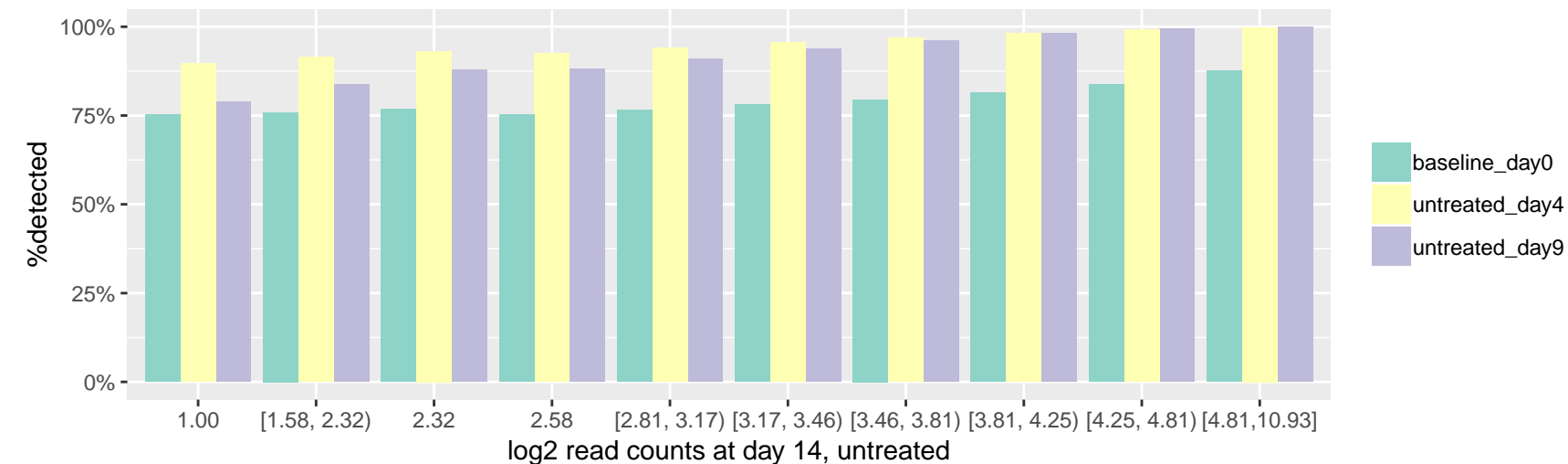

CRISPRwt Clones

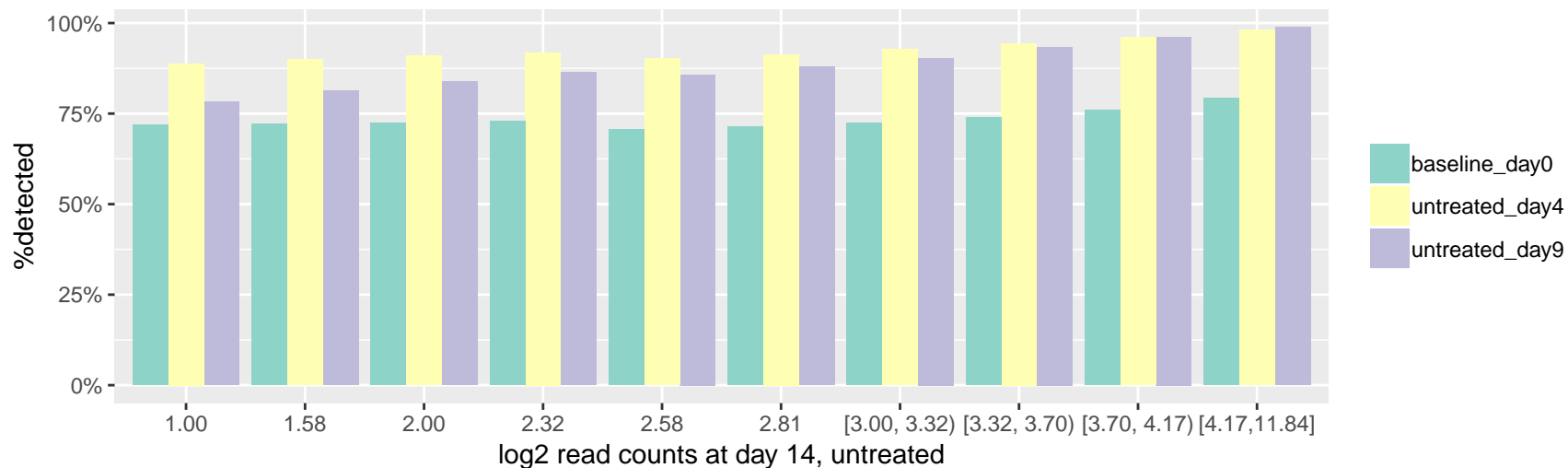

CRISPRwt Cloner

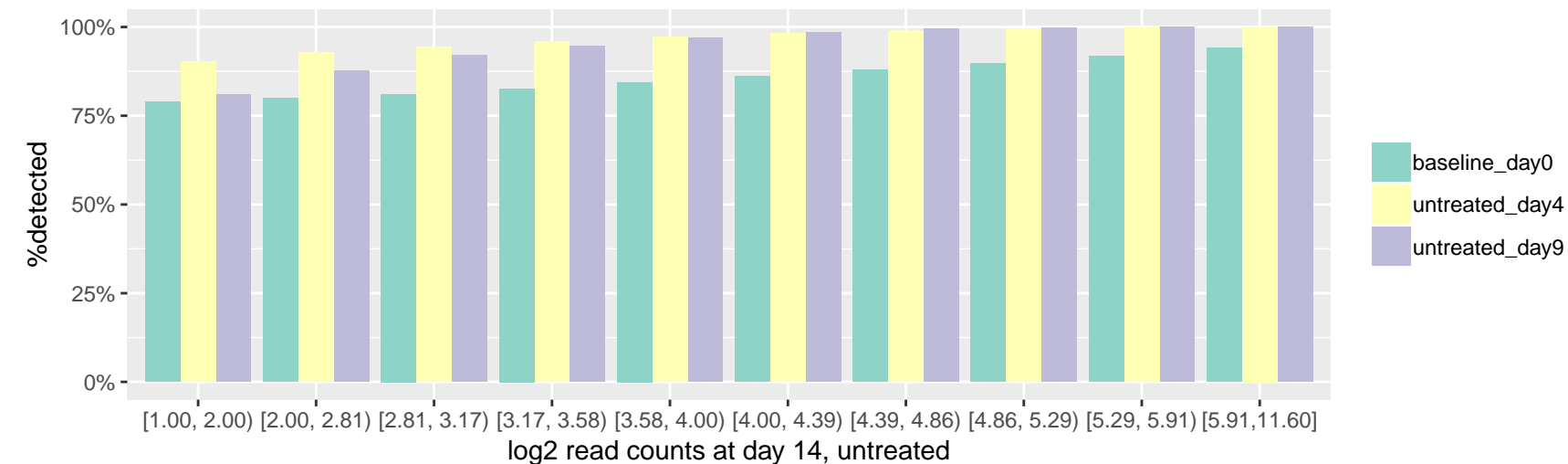

Supplement: Supplementary file 10 — Figure S5. Reproducibility of the sgRNA-barcode pairs across time points of the untreated condition. The x-axis shows 10 bins of sgRNA-barcode pairs that were built based on the quantiles of their log2 read counts distribution at day 14 for each clonal population in the untreated condition. The y-axis shows the proportion of pairs within each bin that were also detected (at least one read count) at baseline/day0, day 4, and day 9. (PDF 7 kb) [file 12864_2019_5480_MOESM10_ESM.pdf]
