# Supplementary material for: Tracing cellular heterogeneity in pooled genetic screens via multi-level barcoding
Source: BMC Genomics. 2019 Feb 6;20:107. doi: 10.1186/s12864-019-5480-0 (PMC6364396; doi:10.1186/s12864-019-5480-0)
Supplement: Supplementary file 9 — Figure S4. A. Distribution of the (log2) number of barcodes per sgRNA. B. Plot of the density of barcode log2 read count distribution per clonal population and CRISPR system. C. Plot of the density of log2 read count distribution of the three built-in replicates for each clonal population and CRISPR system. D. Scatter plots of sgRNA normalized log2 read counts, at baseline (day 0), between three built-in replicates made by randomly splitting barcodes into three groups for each sgRNA. Spearman rank correlation values between replicates are indicated. (PDF 6104 kb) [file 12864_2019_5480_MOESM9_ESM.pdf]

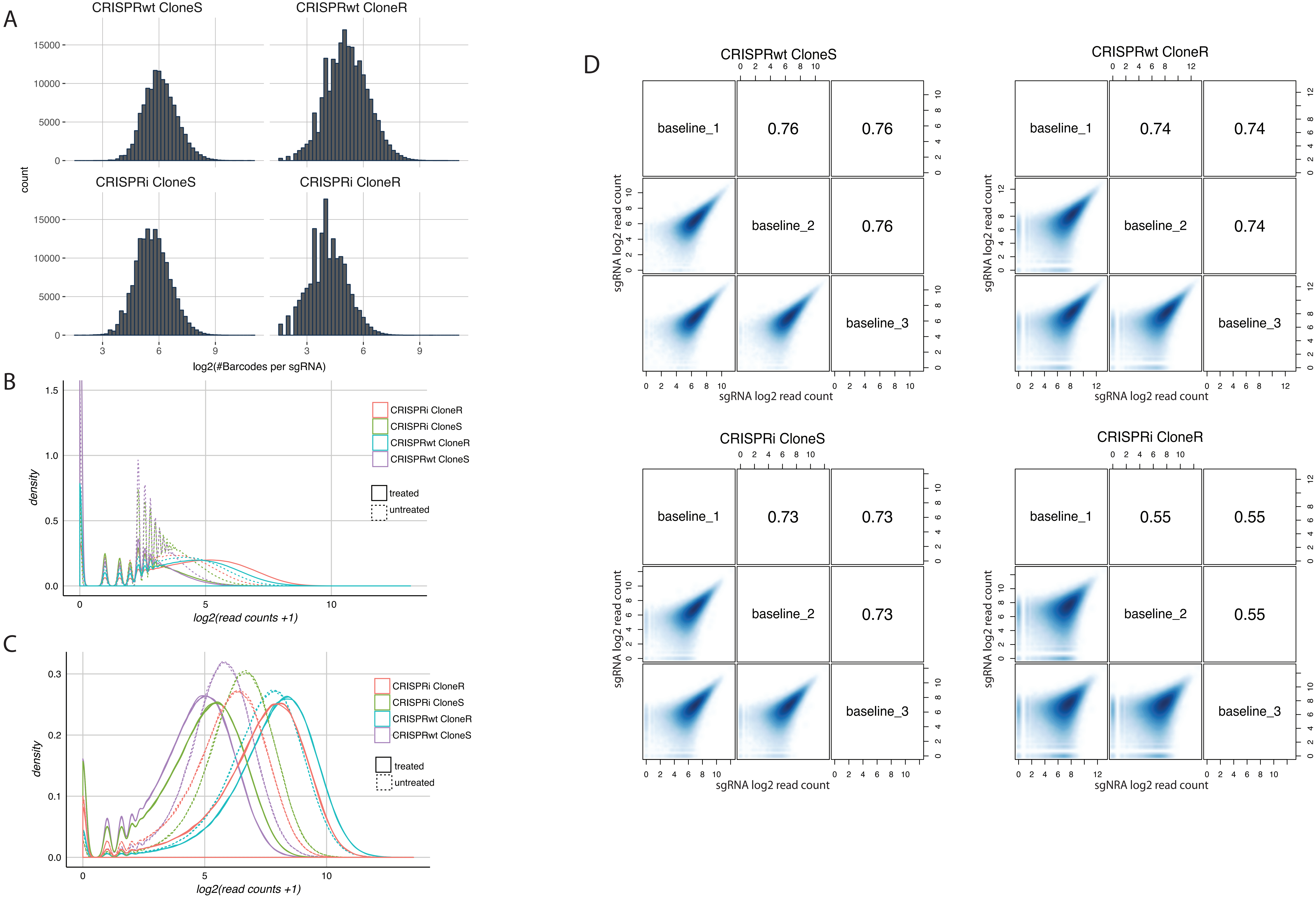
A
CRISPRwt CloneS
CRISPRwt CloneR
CRISPRi CloneS
CRISPRi CloneR
count
log2(#Barcodes per sgRNA)
B
density
log2(read counts +1)
CRISPRi CloneR
CRISPRi CloneS
CRISPRwt CloneR
CRISPRwt CloneS
treated
untreated
C
density
log2(read counts +1)
CRISPRi CloneR
CRISPRi CloneS
CRISPRwt CloneR
CRISPRwt CloneS
treated
untreated
D
CRISPRwt CloneS
baseline_1
0.76
0.76
baseline_2
0.76
baseline_3
sgRNA log2 read count
sgRNA log2 read count
CRISPRwt CloneR
baseline_1
0.74
0.74
baseline_2
0.74
baseline_3
sgRNA log2 read count
sgRNA log2 read count
CRISPRi CloneS
baseline_1
0.73
0.73
baseline_2
0.73
baseline_3
sgRNA log2 read count
sgRNA log2 read count
CRISPRi CloneR
baseline_1
0.55
0.55
baseline_2
0.55
baseline_3
sgRNA log2 read count
sgNRA log2 read count
